# MBGD update 2015: microbial genome database for flexible ortholog analysis utilizing a diverse set of genomic data

**DOI:** 10.1093/nar/gku1152

**Published:** 2014-11-14

**Authors:** Ikuo Uchiyama, Motohiro Mihara, Hiroyo Nishide, Hirokazu Chiba

**Affiliations:** 1 Laboratory of Genome Informatics, National Institute for Basic Biology, National Institutes of Natural Sciences, Nishigonaka 38, Myodaiji, Okazaki, Aichi 444-8585, Japan; 2 Data Integration and Analysis Facility, National Institute for Basic Biology, National Institutes of Natural Sciences, Nishigonaka 38, Myodaiji, Okazaki, Aichi 444-8585, Japan; 3 Dynacom Co., Ltd. 5-1-27, Onoedori, Chuo-ku, Kobe, Hyogo 651-0088, Japan

## Abstract

The microbial genome database for comparative analysis (MBGD) (available at http://mbgd.genome.ad.jp/) is a comprehensive ortholog database for flexible comparative analysis of microbial genomes, where the users are allowed to create an ortholog table among any specified set of organisms. Because of the rapid increase in microbial genome data owing to the next-generation sequencing technology, it becomes increasingly challenging to maintain high-quality orthology relationships while allowing the users to incorporate the latest genomic data available into an analysis. Because many of the recently accumulating genomic data are draft genome sequences for which some complete genome sequences of the same or closely related species are available, MBGD now stores draft genome data and allows the users to incorporate them into a user-specific ortholog database using the MyMBGD functionality. In this function, draft genome data are incorporated into an existing ortholog table created only from the complete genome data in an incremental manner to prevent low-quality draft data from affecting clustering results. In addition, to provide high-quality orthology relationships, the standard ortholog table containing all the representative genomes, which is first created by the rapid classification program DomClust, is now refined using DomRefine, a recently developed program for improving domain-level clustering using multiple sequence alignment information.

## INTRODUCTION

During the last two decades, microbial genome data have been accumulated at an accelerating pace, and now approximately 3000 complete sequences covering species across a wide taxonomic range are available. These complete genomic data are a valuable resource for investigating microbial diversity by comparative analysis. However, recent advances in next-generation sequencing technologies have drastically reduced sequencing costs and are now changing the style of microbial genome sequencing projects (1); genomic sequences of tens or even hundreds of strains of a particular species can be simultaneously determined (2). A majority of genomic sequences are now released as draft genome sequences, for which some complete genome sequences of the same or closely related species are available. The RefSeq microbial genome database has been recently reorganized to accommodate this situation, where genomes of the same species are collected into a directory and representative genomes are selected for each species after several quality checks (3). To effectively utilize these data in medical, industrial or other applications, a comparative genomics approach is essential.

MBGD is a microbial genome database for comparative analysis based on ortholog analysis (4). The central function of MBGD is to provide a set of ortholog groups (an ortholog table) constructed among a specified set of genomes using a hierarchical clustering program, DomClust (5). Remarkable features of MBGD in comparison to the related databases allowing microbial genome comparison such as IMG (6), MicrobesOnline (7), eggNOG (8) and OMA (9) is its flexibility in ortholog analysis. In addition to the standard (default) ortholog table that contains a representative set of genomes covering the entire taxonomic range, MBGD provides a taxon-specific ortholog table constructed for each major taxon in each taxonomic rank (superkingdom, phylum, class, order, family, genus and species) (10). Moreover, MBGD allows the users to upload their own genome sequences for incorporation into the ortholog analysis by dynamically executing ortholog clustering (MyMBGD functionality) (11). By virtue of these features, MBGD provides the users a flexible environment for microbial comparative genome analysis, suitable for comparisons of both closely and distantly related genomes.

To date, MBGD incorporates only complete genome data. This strategy preserves the quality of ortholog classification because in incomplete genome data, the presence/absence of orthologs of given genes generally cannot be correctly determined, impeding correct ortholog identification. Moreover, incorporating draft genome data into all-against-all comparative analysis is not realistic because they accumulate much more rapidly than complete genome data and, thus, will quickly exhaust our computational resources. However, the recent accumulation of draft genome sequence data described above motivated us to incorporate these draft data into MBGD to facilitate comparative analysis of these vast amounts of data. Our strategy for using draft genome data is two-fold: (i) to expand the standard ortholog table by adding only taxonomically unique draft genomes and (ii) to allow the users to incorporate draft genomes into ortholog analysis on an on-demand basis via MyMBGD functionality. For the latter purpose, we have enhanced the MyMBGD interface to allow the users to incorporate not only their uploaded genomes but also the specified draft genome data stored in MBGD.

The quality of ortholog classification is another important issue common to all ortholog databases. One of the prominent features of MBGD is that the ortholog classification is at the domain level for the proper classification of proteins that have experienced domain fusion or fission events, as in the Clusters of Orthologous Group (COG) database where classification was performed with manual modification (12). Here, the domain-level classification is created using the clustering procedure based on local alignment-based sequence similarities rather than using existing domain databases such as Pfam, as in some other databases or programs (13,14). In MBGD, DomClust (5) efficiently performs domain-level ortholog classification using all-against-all protein sequence similarities, but sometimes it generates problematical classifications because domain-level classification is not always easy when only pairwise similarity relations are used. Recently, we developed a program to refine domain-level ortholog classification using multiple sequence alignment information, named DomRefine (15). We now use DomRefine to refine the standard ortholog table.

Here, we describe the recent enhancement of MBGD focusing on a novel data-processing strategy to accommodate the large amount of genomic data while preserving the quality of ortholog classification.

## DATA SOURCES

MBGD collects genomic data of both prokaryotic microbes and eukaryotic microbes (fungi and protozoa) along with four multicellular eukaryotes (human, fruit fly, *Caenorhabditis elegans* and *Arabidopsis thaliana*) for comparison purposes. For prokaryotic complete genomes, we combined three data sources, Gene Trek in Prokaryote Space (GTPS) (16) from DDBJ, GenBank (17) and RefSeq (3) from NCBI, as previously described (10). For other data, we used the RefSeq genome database available at the NCBI Genomes ftp site. We used the data in a Bacteria_DRAFT directory for prokaryotic draft genomes and those in Fungi and Protozoa directories for eukaryotic microbial genomes. In addition, for eukaryotic draft genomes, we used the information in an ASSEMBLY_REPORTS directory to obtain the corresponding RefSeq entries. Draft genome data are discarded if the number of contigs is >2000. The latest MBGD release (2014-02) includes 2823 complete genomes and 5566 draft genomes, including 46 complete and 114 draft eukaryotic genomes.

## OVERVIEW OF THE DATA-PROCESSING PROCEDURE

**Figure 1.**
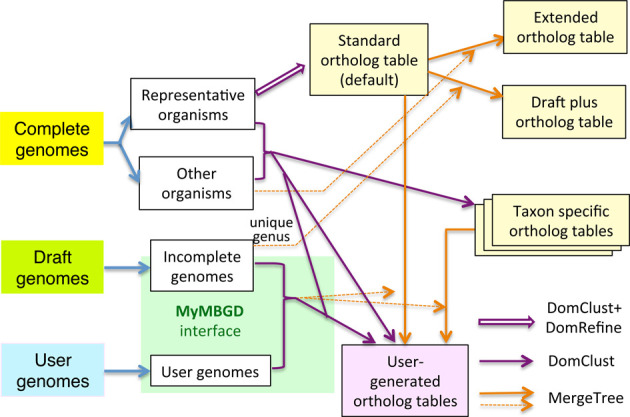
Overview of the data construction procedure in MBGD. Precomputed ortholog tables are colored in light yellow and user-generated ortholog tables are colored in light pink. Three methods (DomClust followed by DomRefine, DomClust only and MergeTree) to create these ortholog tables are shown with different arrows. MergeTree is a program for adding genomes incrementally to an existing ortholog table (base cluster), and thus is represented by two arrows: a base cluster is shown by a solid arrow and an added genome is shown by a broken arrow.

The current data construction procedure in MBGD is summarized in Figure 1. In MBGD, a representative organism set is defined by selecting one genome from each genus in the increasing order of release date from the complete genome data. The standard ortholog table is created from these representative genomes using DomClust followed by the DomRefine procedure (see below). The genes in the other genomes are then assigned to one of the ortholog groups in the standard ortholog table to create an extended ortholog table that contains all the complete genomes stored in MBGD using an incremental procedure implemented in the MergeTree program (10), which incrementally adds genomes to a given ortholog table (named base cluster).

In the new version, we also created another extended ortholog table named ‘draft-plus’ by adding draft genomes belonging to unique genera not included in the representative organism set to the standard ortholog table. In addition to these standard and extended ortholog tables, taxon-specific ortholog tables are generated for each taxonomic group using DomClust.

Besides these precomputed ortholog tables, MBGD allows the users to dynamically create their own ortholog tables. There are two routes for the users to generate new ortholog tables. If the target genomes are all published complete genomes, MBGD executes clustering using the precalculated all-against-all similarities stored in MBGD. In contrast, if the target genomes include user-uploaded or draft genomes, all-against-all similarities including these genomes are dynamically calculated. For the latter route, the enhanced MyMBGD interface enables the users to create a user-generated ortholog table (see below).

## STANDARD ORTHOLOG TABLE REFINED BY DomRefine

**Figure 2.**
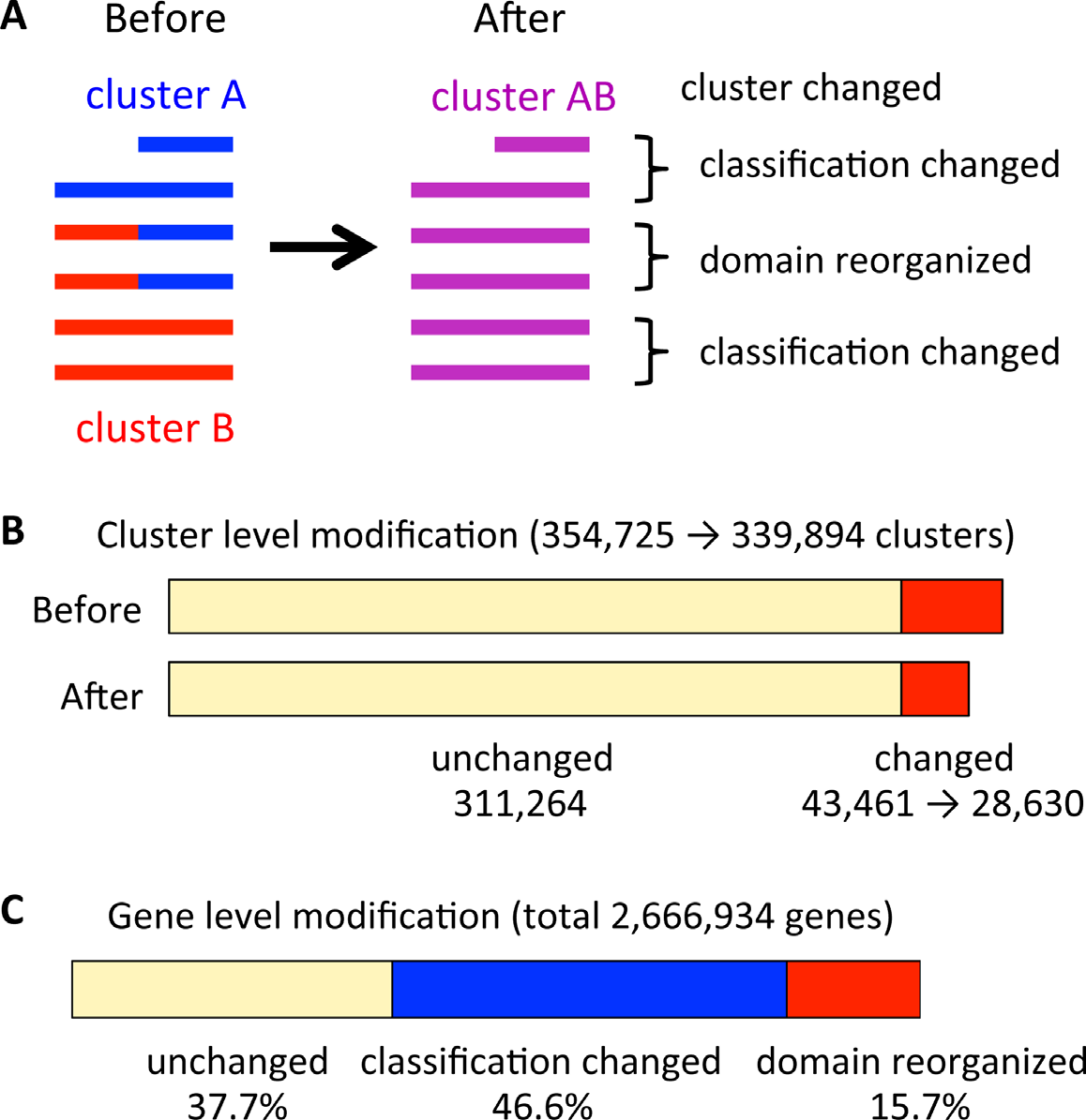
Modification of the domain-level classification in the standard ortholog table by DomRefine. (**A**) An example of modification by DomRefine. Here, two clusters A and B are merged into a new cluster AB. In this case, the number of clusters is reduced from two to one (cluster-level modification) and the numbers of domain-reorganized genes and of classification-changed genes are two and four, respectively (gene-level modification). (**B**) The effect of cluster-level modification by DomRefine. (**C**) The effect of gene-level modification by DomRefine.

Although MBGD can provide various specialized ortholog tables, the standard (default) ortholog table covering the entire taxonomic diversity is the most important for general use. Unfortunately, however, preserving the quality of ortholog classification generally becomes more difficult when a more diverse set of genomes is to be incorporated. Thus, improving the domain-level classification in the standard ortholog table is a critical issue. In this release, the DomRefine pipeline was used to improve the domain-level classification generated by DomClust to create a refined version of the standard ortholog table. DomRefine takes DomClust output as input, and for each pair of domain-level ortholog groups that are adjacent in at least one common protein, it constructs a multiple sequence alignment containing both groups and tries to modify the domain organization by maximizing the sum of the domain-level alignment scores (domain-specific sum-of-pairs or DSP score) of the multiple sequence alignment (15) (Figure 2A). During this optimization procedure, DomRefine also tries to split a cluster into smaller groups according to the phylogenetic gene tree constructed from the multiple sequence alignment (15).

After the refinement, the number of clusters changed from 354,725 in the DomClust output to 339,894 in the DomRefine output, among which 43,461 DomClust clusters were reorganized into 28,630 DomRefine output (Figure 2B). However, the effect of refinement is greater than it appears here because the majority of the clusters are singleton or very small clusters. At the gene level, domain reorganization occurred in 15.7% and classification was changed in 46.6% of the genes (Figure 2C).

**Figure 3.**
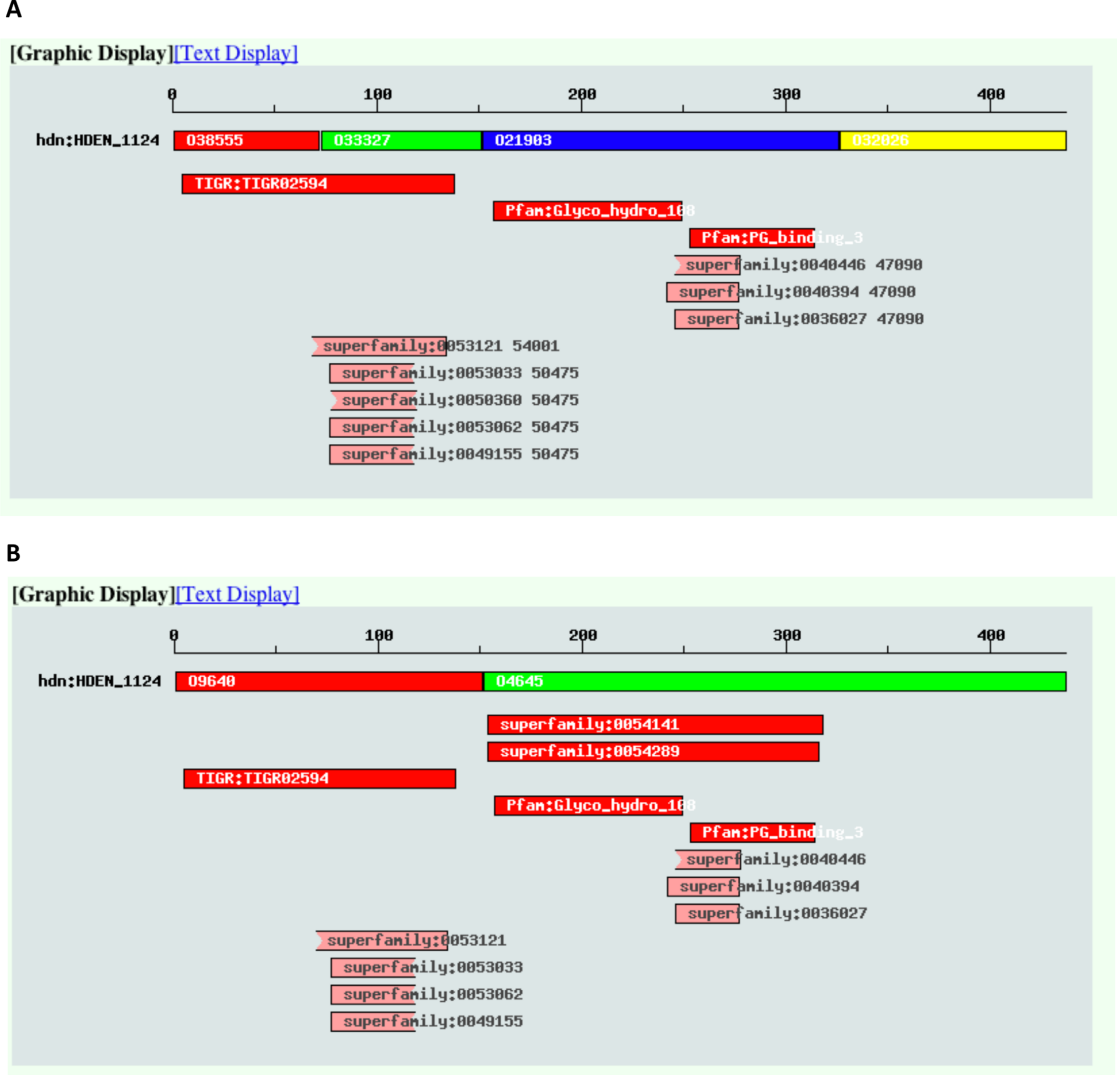
An example of domain reorganization by DomRefine. Shown are the domain organizations of the gene entry hdn:HDEN_1124 in the MBGD gene information pages in the version 2014-01 (**A**; without refinement) and 2014-02 (**B**; after refinement). In each figure, the first line indicates the domain organization in MBGD and the subsequent lines indicate the domains identified by HMMER search against the databases included in InterPro (19).

Classification quality can be evaluated by comparison with a reference classification. Here, we use the TIGRFAMs database (18) as a reference classification and the number of one-to-one corresponding clusters with TIGRFAMs (*N_TIGR_^1to1^*) as a measure of agreement, as in our previous work (15). Then *N_TIGR_^1to1^* is increased from 1228 in DomClust output to 1328 in DomRefine output. An example of a protein in which domain reorganization occurred is shown in Figure 3. Here, the domain organizations of the gene entry hdn:HDEN_1124 (hypothetical protein from *Hyphomicrobium denitrificans* ATCC 51888) before and after refinement are shown, together with the domain search result using InterPro (19). This gene was originally split into four domains, but after refinement the first and last pairs of domains are respectively merged into two domains (Figure 3) and now the N-terminal half has a one-to-one correspondence with a TIGRFAMs entry TIGR02594 (a conserved protein family with unknown function).

Previously, MBGD did not store the multiple sequence alignment information for each ortholog group but generated it dynamically upon request. Because DomRefine generates a multiple sequence alignment and a phylogenetic tree for each ortholog group as by-products, MBGD now stores these data and returns them upon request for the standard ortholog group. This feature can shorten the response time for returning an alignment, particularly for a large ortholog group.

## THE DRAFT-PLUS STANDARD ORTHOLOG TABLE

A part of the draft genomes are used to extend the standard ortholog table to create the draft-plus ortholog table. For this purpose, a set of draft genomes, each of which belongs to a unique genus not included in the representative organism set, is added to the standard ortholog table using the MergeTree program. In the release 2014-02, the number of representative genomes in the standard table is 742 and the draft-plus table includes additional 427 draft genomes. This increases the taxonomical diversity of the target organisms; the number of unique taxa increases by approximately 30% at the family, order and class levels and increases even at the phylum level (Table 1). Among the 427 draft genomes, 72 are eukaryotes, whereas 37 of the 742 genomes are eukaryotes in the standard ortholog table. Thus, eukaryotic microbial genomes are substantially increased in the draft-plus ortholog table, reflecting the release of the majority of eukaryotic genomes as draft sequences.

**Table 1.** Taxonomic diversity of the standard and the draft-plus ortholog tables

	Standard (742 genomes)	Standard + draft (427 genomes)
Phylum	50	57
Class	63	82
Order	133	168
Family	262	336

By default, MBGD uses the standard ortholog table as the default ortholog table, but the users can switch to using the draft-plus table by clicking ‘Draft-plus’ button on the top page. On the other hand, because the extended ortholog table has the same cluster IDs as the original one, the users can see the information of the corresponding draft-plus ortholog group from each ortholog cluster page.

## ENHANCEMENT OF MyMBGD: INCORPORATING DRAFT GENOMES IN ADDITION TO USER GENOMES INTO ORTHOLOG ANALYSIS

The MyMBGD interface allows the users to upload their own genome data to the MBGD server and incorporate them into ortholog analysis by calculating all-against-all similarities between the uploaded genomes and the prestored complete genomes, followed by ortholog clustering (11). The users can now specify the draft genomes prestored in MBGD in addition to the uploaded user genomes as the target of analysis.

There are two ways to incorporate them into the ortholog analysis: creating ortholog groups from scratch using DomClust or adding to an existing (either standard or taxon specific) ortholog table using MergeTree. Overall, the users can choose from the following three modes of analysis: (i) *taxon-specific comparison mode*, where the users first choose a taxon to be analyzed and then specify a set of genomes to be compared within that taxon. In this mode, the users can create an ortholog table either from scratch by choosing arbitrary set of genomes in that taxon or by adding new genomes to a taxon-specific ortholog table; (ii) *mapping on the standard ortholog table mode*, in which selected genomes will be added to the standard ortholog table using MergeTree; and (iii) *free genome selection mode*, which is equivalent to the previous MyMBGD mode, in which the users can freely choose a set of genomes to compare including user genomes, complete genomes and draft genomes and conduct ortholog analysis using DomClust.

**Figure 4.**
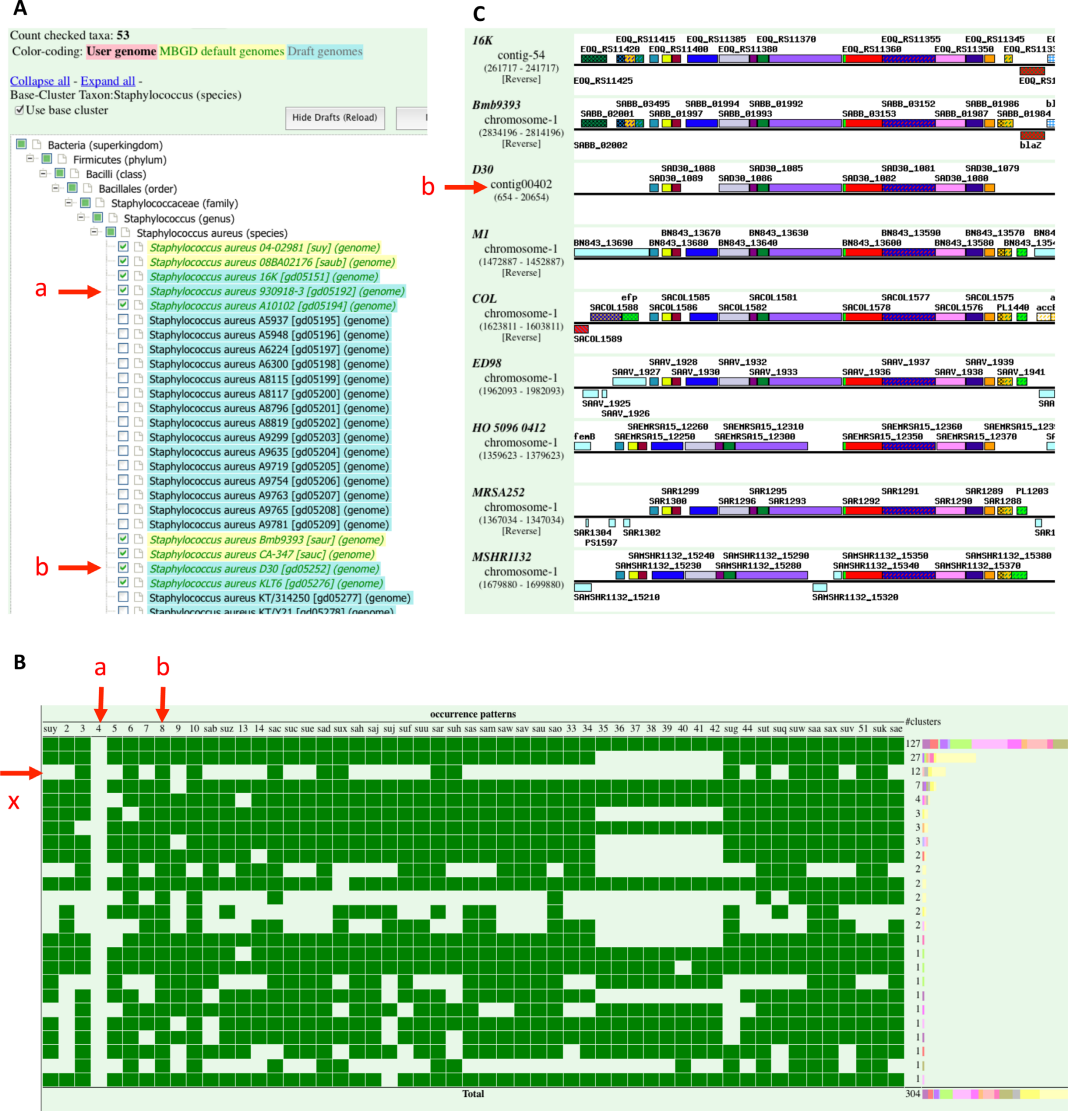
An example session of the MyMBGD analysis, where comparison of *Staphylococcus aureus* genomes was performed focusing on two strains, 930918-3 and D30, which are indicated with ‘a’ and ‘b’, respectively. (**A**) The MyMBGD interface for specifying a set of genomes in taxon-specific comparison mode. Complete genomes are shown in light yellow and draft genomes are shown in light blue. (**B**) Occurrence-pattern display in which ortholog groups that are present in strain D30 and absent in strain 930918-3 are extracted and summarized according to occurrence pattern. The occurrence pattern corresponding to the transposon-like cluster containing the FtsK/SpoIIIE family protein is indicated with ‘x’. (**C**) Genome region map comparison viewer showing gene order conservation around the ortholog group of the FtsK/SpoIIIE family protein. Here, orthologous genes are drawn in the same color and pattern.

Figure 4 shows an example session of the taxon-specific comparison mode in the new MyMBGD interface, where strains of *Staphylococcus aureus* including five draft genomes and 48 complete genomes are specified for comparison (Figure 4A). Here, these draft genomes include strains D30 and 930918-3, which have been investigated as models for nasal carrier and noncarrier strains, respectively (20). *S. aureus* is a versatile human pathogen and its infection in hospitals and the emergence of drug-resistant strains poses a worldwide problem. Comparison of these two strains is interesting in terms of infectious mechanisms because the presence of *S. aureus* in the nose increases the risk of its infection (21). After ortholog clustering among these genomes is completed, occurrence (presence/absence) pattern analysis can be conducted on the cluster map page; in this example, ortholog groups included in the D30 strain but not in the 930918-3 strain are extracted and displayed (Figure 4B). For some ortholog groups in which a user is interested, a detailed analysis can be conducted using any functionality in MBGD. In this example, by selecting a particular occurrence pattern (indicated with ‘x’ in Figure 4B), one can identify a transposon-like cluster carrying several genes including a FtsK/SpoIIIE family protein and TraG protein, which was listed as an interesting observation by the original authors (20). The arrangement of these genes in each chromosome can then be compared on the genome region map comparison viewer (Figure 4C).

## CONCLUSIONS AND FUTURE PERSPECTIVES

By the enhancements introduced here, MBGD now allows the users to incorporate publicly available draft genome data in addition to complete genome data into comparative analysis. This functionality can facilitate the use of the vast amount of sequence data in various studies. The refined version of the standard ortholog table can also provide a good basis for large-scale genomics studies, including the draft genome annotation accomplished in the draft-plus ortholog table created in this study, and possibly for metagenome analysis in future. Given that the accelerated increase in genomic data will continue, we need to continue to seek more efficient ways to process and manage these data. In particular, managing all-against-all similarity relationships is likely to become a critical issue in the near future. Another important issue is to provide more effective data presentation for a large-scale comparative analysis. To overcome the limitations of conventional web browser-based data retrieval, we are also preparing a Resource Description Framework version of MBGD that can be retrieved by a SPARQL query.
